# A study of factors influencing long-term glycemic variability in patients with type 2 diabetes: a structural equation modeling approach

**DOI:** 10.3389/fendo.2023.1216897

**Published:** 2023-07-31

**Authors:** Yuqin Gan, Mengjie Chen, Laixi Kong, Juan Wu, Ying Pu, Xiaoxia Wang, Jian Zhou, Xinxin Fan, Zhenzhen Xiong, Hong Qi

**Affiliations:** ^1^ School of Nursing, Chengdu Medical College, Chengdu, China; ^2^ Clinical Medical College of Chengdu Medical College, First Affiliated Hospital, Chengdu, China; ^3^ Department of Endocrinology and Metabolism, The First Affiliated Hospital of Chengdu Medical College, Chengdu, China; ^4^ Department of Rheumatology and Immunology, The First Affiliated Hospital of Chengdu Medical College, Chengdu, China

**Keywords:** type 2 diabetes mellitus, glycated hemoglobin, glycemic variability, influential factors, structural equation modeling

## Abstract

**Aim:**

The present study aims to utilize structural equation modeling (SEM) to investigate the factors impacting long-term glycemic variability among patients afflicted with type 2 diabetes.

**Method:**

The present investigation is a retrospective cohort study that involved the collection of data on patients with type 2 diabetes mellitus who received care at a hospital located in Chengdu, Sichuan Province, over a period spanning from January 1, 2013, to October 30, 2022. Inclusion criteria required patients to have had at least three laboratory test results available. Pertinent patient-related information encompassing general demographic characteristics and biochemical indicators was gathered. Variability in the dataset was defined by standard deviation (SD) and coefficient of variation (CV), with glycosylated hemoglobin variation also considering variability score (HVS). Linear regression analysis was employed to establish the structural equation models for statistically significant influences on long-term glycemic variability. Structural equation modeling was employed to analyze effects and pathways.

**Results:**

Diabetes outpatient special disease management, uric acid variability, mean triglyceride levels, mean total cholesterol levels, total cholesterol variability, LDL variability, baseline glycated hemoglobin, and recent glycated hemoglobin were identified as significant factors influencing long-term glycemic variability. The overall fit of the structural equation model was found to be satisfactory and it was able to capture the relationship between outpatient special disease management, biochemical indicators, and glycated hemoglobin variability. According to the total effect statistics, baseline glycated hemoglobin and total cholesterol levels exhibited the strongest impact on glycated hemoglobin variability.

**Conclusion:**

The factors that have a significant impact on the variation of glycosylated hemoglobin include glycosylated hemoglobin itself, lipids, uric acid, and outpatient special disease management for diabetes. The identification and management of these associated factors can potentially mitigate long-term glycemic variability, thereby delaying the onset of complications and enhancing patients’ quality of life.

## 1 Introduction

The prevalence of diabetes has increased threefold over the past 30 years (1), making it one of the most prevalent and rapidly growing illnesses. By 2045, it is projected that diabetes will affect 12.2% of the population (783.2 million) worldwide (2). In China, the prevalence rate of diabetes has reached 11.2% since 2015-2017, with type 2 diabetes accounting for over 90% of cases (3). Globally, diabetes is the ninth leading cause of mortality (1), accounting for 4.2 million deaths in 2019 alone (4), which is 2-3 times higher than in non-diabetic individuals (5). Morbidity and mortality in diabetes are mainly caused by cardiovascular disease, diabetic nephropathy, retinopathy, and neuropathy (6). Hospitalization costs for diabetic patients vary widely across different Asian countries and can range from 11% to 75% of per capita income, with three times higher costs for patients with complications (7). In 2019, the global cost of direct treatment for diabetes was estimated to be $760 billion, with China alone spending $109 billion (8). Moreover, hospitalization costs were found to be three times higher for diabetic patients with complications than for those without complications (7).

Glycosylated hemoglobin (HbA_1c_) is widely used as the benchmark for glycemic control and treatment of diabetic patients in clinical settings (3). The primary therapeutic goal for diabetic patients is to maintain blood glucose levels to prevent complications (9). However, diabetes experts agree that the extent of glycemic fluctuations among diabetic patients with similar HbA_1c_ management may affect their risk of complications (10). Numerous studies have established a correlation between glycemic variability (GV) and diabetic complications (11–13), and it has been established that GV is a more clinically significant glycemic control indicator than HbA_1c_ (14). Bergenstal et al. (15) demonstrated that GV refers to the fluctuations in blood glucose levels, primarily reflecting a decline in pancreatic β-cell function and is more detrimental to diabetic patients than persistent and stable hyperglycemia; it encompasses short-term and long-term glycemic variability, with short-term GV being characterized by intra-day or inter-day fluctuations in blood glucose that ultimately result in an increase in HbA_1c_ (16). Long-term glycemic variability refers to the inconsistency of HbA_1c_ values as they fluctuate between peaks and troughs (11). Therefore, optimal glycemic control for diabetic patients involves targeting HbA_1c_ levels while also minimizing HbA_1c_ fluctuation as much as possible (10).

In recent years, long-term glucose variability has garnered considerable attention from experts and clinicians (17). An increasing number of studies utilize it as a primary determinant of glycemic control quality (18). The glucose standard deviation (SD) and coefficient of variation (CV) are currently the most commonly employed measures (18). Recently, novel research has proposed a new metric, the HbA_1c_ variability score (HVS), which calculates the proportion of HbA_1c_ variations exceeding 0.5% from the previous measurement, divided by the total number of HbA_1c_ measurements, to evaluate long-term glucose fluctuations (19, 20).

The degree of fluctuation in HbA_1c_, as opposed to absolute blood glucose levels, is strongly associated with unfavorable outcomes (21). Among diabetic patients, HbA_1c_ variability is a risk factor for mortality and death due to cardiovascular disease (22, 23), and it is independent of HbA_1c_ levels (24). In addition, numerous observational and randomized clinical trial studies have demonstrated that HbA_1c_ variability is linked to the risk of both microvascular and macrovascular complications in diabetes patients, as well as increased occurrences of hypoglycemia (12, 19, 25–27). Furthermore, a meta-analysis revealed that HbA_1c_ variability is connected with the incidence of dementia among diabetic patients (28). Therefore, the degree of HbA_1c_ fluctuation may play a crucial role in the clinical assessment of risks related to complications and mortality.

Given the well-established connection between HbA_1c_ variability and diabetic complications and mortality, reducing HbA_1c_ variability should be a target of glycemic management for diabetic patients (29). To effectively reduce HbA_1c_ variability in clinical practice, it is crucial to comprehend the factors that impact it in order to more successfully control HbA_1c_ variability, delay the onset of complications, lower mortality rates in diabetic patients, and improve disease prognosis and quality of life. However, there is a dearth of trustworthy literature on the factors that influence HbA_1c_ variability. Consequently, the purpose of this study is to perform a retrospective analysis to investigate the present status of HbA_1c_ variability and its influencing factors, as well as to establish a reference basis for clinical concepts on strategies to decrease HbA_1c_ variability in later stages.

## 2 Methods

### 2.1 Study design and participants

Clinical data of patients were obtained from the outpatient system of the Department of Endocrinology at a tertiary care hospital located in Chengdu for the purpose of collecting data from type 2 diabetes mellitus patients who were managed in the outpatient clinic from 2013 to 2022. Inclusion criteria were as follows: (1) meeting the diagnostic and classification criteria for type 2 diabetes according to the 1999 version of the World Health Organization (WHO); (2) age at the time of initial diagnosis was 18 years or older; and (3) at least three laboratory test findings were available. Exclusion criteria comprised: (1) the presence of other serious chronic diseases affecting the heart, liver, kidneys, brain, and lungs at the time of inclusion; (2) missing laboratory test data; (3) missing general data; and (4) presence of other endocrine diseases, such as thyroid, pituitary, and adrenal diseases, at the time of initial diagnosis.

### 2.2 Data collection

The medical records of patients receiving outpatient care at a tertiary hospital in Chengdu’s endocrinology department were reviewed to extract clinical data, including demographic variables such as gender and age, as well as uric acid levels, and other variables including triglycerides (TG), total cholesterol (TC), high-density lipoprotein (HDL), low-density lipoprotein (LDL), blood glucose levels, and glycated hemoglobin (HbA_1c_). The patients were also categorized based on their enrollment in the Outpatient Special Disease Management program, which involved regular HbA_1C_ measurements, health education, medication prescriptions, and timely adjustments by a diabetes specialist. By organizing and analyzing the data, the variability and correlation values of each variable were determined.

### 2.3 Statistical analysis

The study data were exported from the EpiData (Chinese version) management software and subjected to analysis using IBM SPSS 26.0 and IBM SPSS AMOS 28.0 software. The Origin 2022 software was utilized for graphing purposes. Quantitative data were expressed in terms of mean ± standard deviation, while qualitative data were expressed as count and percentage (%). Linear regression analysis was employed to evaluate the impact of type 2 diabetes data on long-term glycemic variability. Additionally, structural equation modeling using AMOS 28.0 software was performed to identify the factors influencing long-term glycemic variability, and analyzed the power with power4SEM program. The goodness of fit index (GFI), incremental fit index (IFI), comparative fit index (CFI), standardized fit index (NFl), relative fit index (RFI), and non-normalized fit index (TLI) were used to evaluate the model fit. The values of IFI, CFI, NFI, RFI, and TLI were all greater than 0.9, while root mean square error of approximation (RMSEA) was less than 0.08, and χ2/df was less than 3 (30). Statistical significance was set at P<0.05.

### 2.4 Ethical considerations

The study was approved by the Ethics Committee of First Affiliated Hospital of Chengdu Medical College (approval no. 2021-07), and it was carried out in accordance with the Code of Ethics of the World Medical Association (Declaration of Helsinki).

## 3 Result

### 3.1 Participant characteristics

This study comprised 369 subjects, the majority of whom were 40 years of age or older (95.7%). They had a follow-up duration of at least 4 years for diabetes mellitus management (69.6%), and were treated as outpatient special diseases (69.9%). A considerable proportion of subjects had comorbidities (83.7%) and multiple complications (85.1%). **Table 1** provides a comprehensive summary of the findings.

**Table 1:** Sociodemographic characteristics of the study subjects (N=369).

Variables	Category	Sample n (%)
Gender	Man	190 (51.5%)
	Woman	179 (48.5%)
Age	≤40years	16 (4.3%)
	41-60years	162 (43.9%)
	61-80years	176 (47.7%)
	≥81years	15 (4.1%)
Follow-up time	1-3years	112 (30.4%)
	4-6years	124 (33.6%)
	≥7years	133 (36.0%)
Outpatient special disease management	Yes	258 (69.9%)
	No	111 (30.1%)

### 3.2 HbA_1c_ variant distribution characteristics

**Figure 1:**
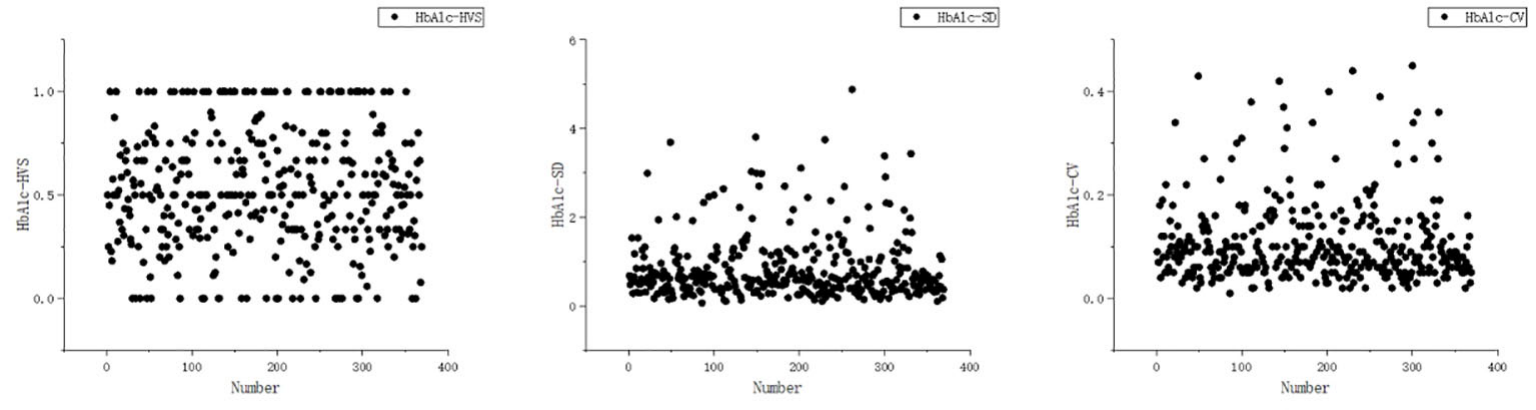
Characteristics of HbA_1c_ variant distribution in T2DM patients.

In this study, the mean and standard deviation of HbA_1C_-HVS were 0.529 ± 0.285, HbA_1C_-SD were 0.833 ± 0.712, and HbA_1C_-CV were 0.109 ± 0.079. Detailed results can be found in **Table 2**. Specifically, 64.50% of HbA_1C_-HVS values ranged from 25.00% to 75.00%, 69.11% of HbA_1c_-SD values ranged from 0.35 to 1.50, and 58.14% of HbA_1c_-CV values were between 0.05 and 0.15. The distribution of each data type among different patients is illustrated in **Figure 1**.

**Table 2:** Descriptive statistics of HbA_1c_ variants in T2DM patients.

	Mean	Standardized	Skewness	Peakedness
HbA_1C_-HVS	0.529	0.285	0.016	-0.702
HbA_1c_-SD	0.833	0.712	2.235	5.969
HbA_1C_-CV	0.109	0.079	1.977	4.411

### 3.3 Biochemical index variability in T2DM patients: descriptive statistics

In this investigation, the mean values and standard deviations for the variability of biochemical markers were determined as follows: UA-SD (51.509 ± 33.448), UA-CV (0.160 ± 0.106), TG-SD (0.977 ± 2.774), TG-CV (0.382 ± 0.349), TC-SD (0.677 ± 0.483), TC-CV (0.165 ± 0.088), LDL-SD (0.578 ± 0.548), LAL-CV (0.268 ± 0.154), HDL-CV (0.139 ± 0.196), HDL-SD (0.354 ± 3.509). Further elaboration of the results can be found in **Table 3**.

**Table 3:** Biochemical index variability in T2DM patients: descriptive statistics.

	Mean	Standardized	Skewness	Peakedness
UA-SD	51.509	33.448	2.361	8.288
UA-CV	0.160	0.106	3.342	17.902
TG-SD	0.977	2.774	10.457	141.382
TG-CV	0.382	0.349	4.647	29.899
TC-SD	0.677	0.483	3.639	21.709
TC-CV	0.165	0.088	1.433	3.354
LDL-SD	0.578	0.548	10.017	145.566
LAL-CV	0.268	0.154	2.964	19.933
HDL-CV	0.139	0.196	12.419	186.634
HDL-SD	0.354	3.509	19.136	367.086

### 3.4 One-way logistic regression analysis of factors influencing HbA_1c_ variants in T2DM patients

In this investigation, it was observed that specific outpatient disease management, changes in uric acid levels, average triglyceride and total cholesterol levels, variability of total cholesterol and LDL, as well as baseline and recent glycation, were significant contributing factors to the variation in HbA_1C_ among patients with type 2 diabetes (P<0.05). The results of the study are presented in **Table 4**, while **Table 5** provides the details of the variable assignments.

**Table 4:** Linear regression analysis of risk factors for HbA_1C_ variants in patients with type 2 diabetes.

Dependent variable	Independent variable	β	standardized coefficient β	t	P-Value	β Percentile95%CI		
						Lower	Upper	R^2^
HbA_1C_-HVS	Outpatient special disease management	13.165	0.212	4.152	<0.001	6.930	19.401	0.045
	UA-SD	0.179	0.210	4.105	<0.001	0.093	0.264	0.044
	UA-CV	58.687	0.219	4.294	<0.001	31.813	85.561	0.048
	TG-mean	2.750	0.163	3.161	0.002	1.039	4.460	0.027
	TC-mean	5.453	0.166	3.230	0.001	2.133	8.772	0.028
	TC-CV	52.975	0.164	3.179	0.002	20.206	85.744	0.028
	TC-SD	12.713	0.215	4.222	<0.001	6.792	18.635	0.046
	LDL-SD	6.886	0.132	2.555	0.011	1.586	12.187	0.017
	Baseline HbA_1C_	5.915	0.383	7.950	<0.001	4.452	7.378	0.147
	Recent HbA_1C_	7.795	0.337	6.865	<0.001	5.562	10.028	0.114
HbA_1C_-SD	Outpatient special disease management	0.390	0.251	4.974	<0.001	0.236	0.544	0.063
	UA-SD	0.007	0.334	6.778	<0.001	0.005	0.009	0.111
	UA-CV	2.124	0.317	6.412	<0.001	1.473	2.776	0.101
	TG-mean	0.059	0.140	2.708	0.007	0.016	0.102	0.020
	TC-mean	0.116	0.141	2.737	0.006	0.033	0.199	0.020
	TC-SD	0.351	0.238	4.699	<0.001	0.204	0.498	0.057
	TC-CV	1.949	0.241	4.767	<0.001	1.145	2.753	0.058
	LDL-SD	0.251	0.193	3.77	<0.001	0.120	0.382	0.037
	LDL-CV	0.609	0.132	2.555	0.011	0.140	1.078	0.016
	Baseline HbA_1C_	0.270	0.702	18.894	<0.001	0.242	0.298	0.493
	Recent HbA_1C_	0.171	0.297	5.969	<0.001	0.115	0.228	0.088
HbA_1C_-CV	Outpatient special disease management	0.039	0.229	4.507	<0.001	0.022	0.056	0.052
	UA-SD	0.001	0.286	5.723	<0.001	0	0.001	0.082
	UA-CV	0.182	0.247	4.884	<0.001	0.109	0.256	0.061
	TG-mean	0.006	0.127	2.453	0.015	0.001	0.011	0.016
	TC-mean	0.010	0.108	2.077	0.038	0.001	0.019	0.012
	TC-SD	0.032	0.195	3.807	<0.001	0.015	0.048	0.038
	TC-CV	0.188	0.210	4.125	<0.001	0.098	0.277	0.044
	LDL-SD	0.023	0.160	3.096	0.002	0.008	0.037	0.025
	LDL-CV	0.057	0.111	2.144	0.033	0.005	0.108	0.011
	Baseline HbA_1C_	0.025	0.580	13.651	<0.001	0.021	0.028	0.337
	Recent HbA_1C_	0.012	0.189	3.680	<0.001	0.006	0.018	0.036

**Table 5:** Assignment of factors affecting patients with T2DM.

	Variables	Variable assignment
X1	Gender	Man=1,Woman=2
X2	Age	≤40years=1;41-60years=2;61-80years=3;≥81years=4
X3	Follow-up time	1-3years=1;4-6years=2;≥7years=3
X4	Outpatient special disease management	YES=1;NO=2

### 3.5 Structural equation model

**Figure 2:**
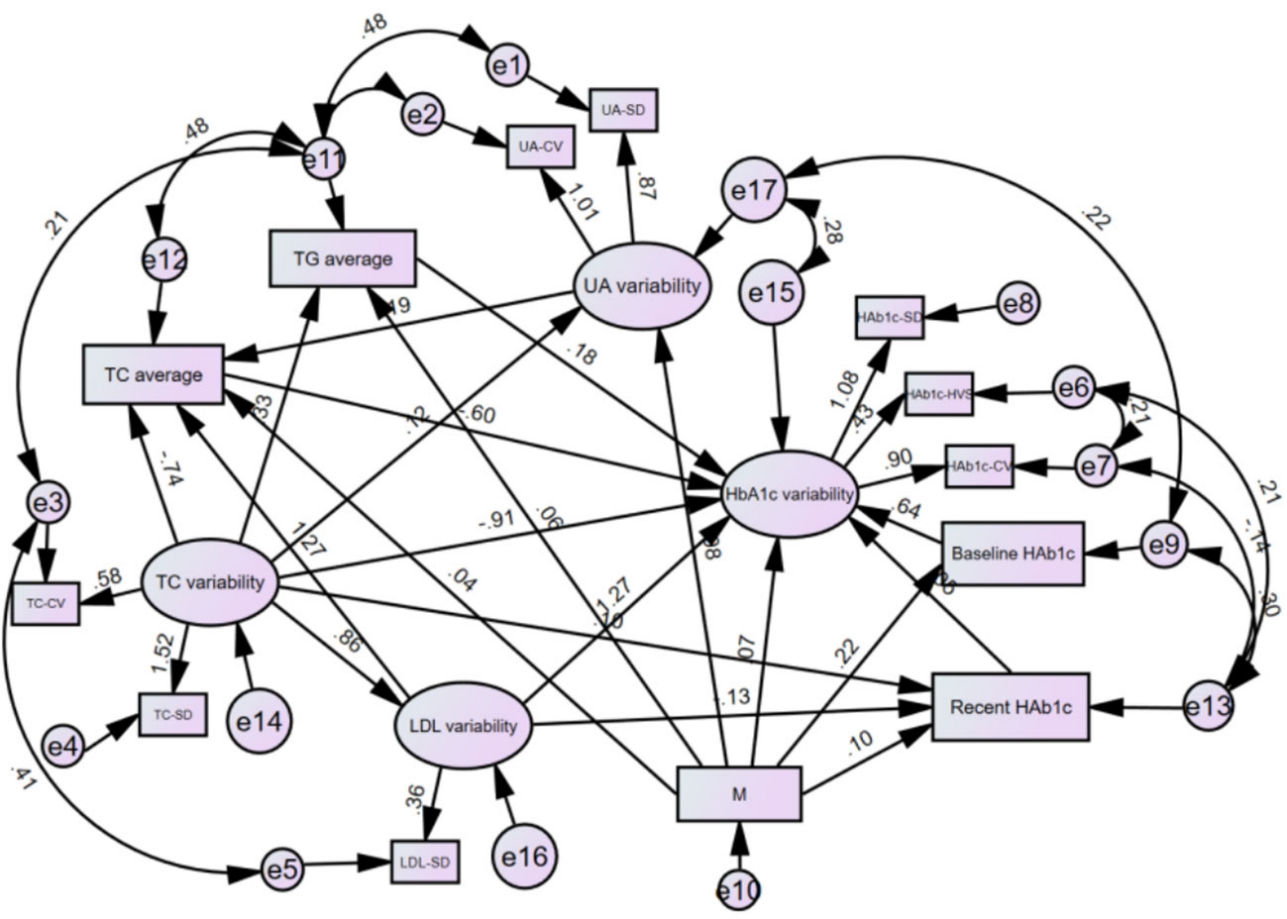
Modified model of structural equations for HbA_1c_ variants in T2DM patients. M.Outpatient sppecial disease management. HbA_1c_ Glycated hemoglobin; UA, Uric acid; TG, Triglycerides; TC, Total cholesterol; LDL, Low-density lipoprotein; CV, Coefficient of Variation; SD, Standard Deviation; HVS, HbA_1c_ variability score.

**Figure 3:**
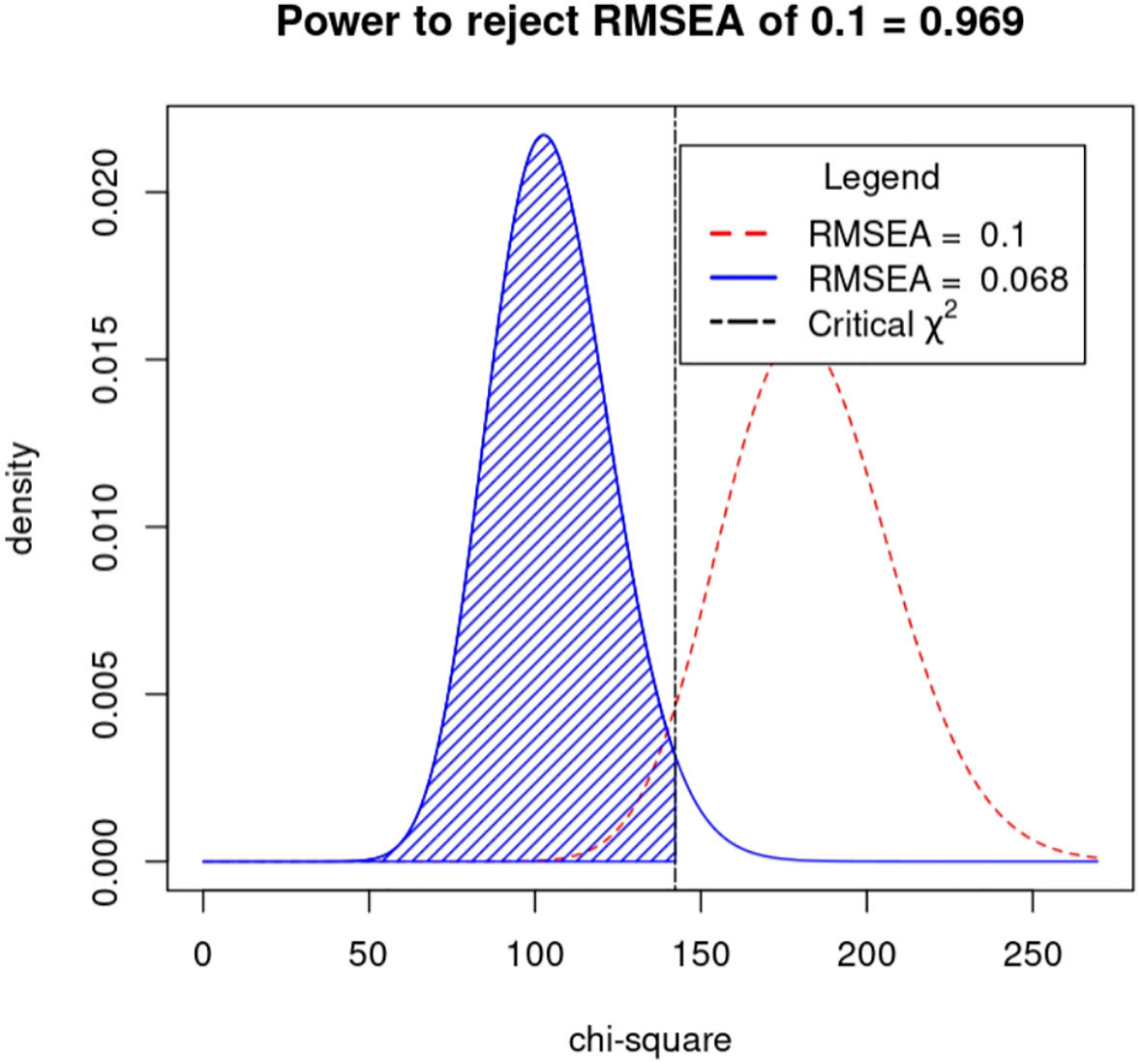
Structural equation modeling study power.

Drawing on the literature on variability in HbA_1C_ levels, the variables that demonstrated a significant impact on such variability were integrated into a structural equation model while taking into account the study’s objectives and domain-specific knowledge. The resulting model incorporated five observed variables - namely, outpatient-specific disease management, mean triglyceride, mean total cholesterol, baseline HbA_1C_, and recent HbA_1C_- along with four latent variables: uric acid variability, total cholesterol variability, LDL variability, and HbA_1c_ variability. The final structural equation model is illustrated in **Figure 2**. The goodness-of-fit indices, which included a χ2/df of 2.698:3, RMSEA of 0.068:0.08, GFI of > 0.9, AGFI of 0.9, NFI of > 0.9, RFI of > 0.9, IFI of > 0.9, TLI of > 0.9, and CFI of > 0.9, revealed a good fit for the model. The model specific fits are detailed in **Table 6**. The power4SEM program and calculated the study efficacy of 0.969 for this study based on the study results RMSEA=0.068, DF=39, N=369, α=0.05. Further details are available in **Figure 3**.

**Table 6:** Evaluation results of the best fit of the optimal model.

Adaptation index	χ2/df	GFI	NFI	RFI	IFI	TLI	CFI	RMSEA
Reference value	<3.00	>0.9	>0.9	>0.9	>0.9	>0.9	>0.9	<0.08
Model test value	2.698	0.957	0.971	0.942	0.981	0.963	0.981	0.068

### 3.6 Effect analysis of structural equation model variables

The present investigation discovered that TC-mean exerted a negative direct impact on HbA_1c_ variability (β=-0.600, P=0.028). Baseline HbA_1C_ exhibited the strongest positive direct influence on HbA_1C_ variability (β=0.644, P=0.001), while TG-mean had a positive direct effect on HbA_1C_ variability (β=0.176, P=0.042) and outpatient specific disease management had a positive direct effect on HbA_1C_ variability (β=0.071, P=0.039). Further details are available in **Table 7**.

**Table 7:** Direct, indirect and total effects of each influencing factor on long-term blood glucose variability.

Influence Factors	Indirect effects	Direct effects	total effects	P-Value
Baseline HbA_1C_	—	0.644	0.644	<0.001
TC-mean	—	-0.600	-0.600	0.028
LDL-Variation	-0.766	1.271	0.505	0.072
DOPD	0.128	0.071	0.199	0.039
TG-mean	—	0.176	0.176	0.042
UA-Variation	-0.112	—	-0.112	—
Recent HbA_1C_	—	0.049	0.049	0.230
TC-Variation	0.927	-0.907	0.020	0.093

“–” Indicates no effect.

## 4 Discussion

In this study, the aim was to investigate the association between HbA_1C_ variability and outpatient special disease management, uric acid, total cholesterol, triglycerides, HDL cholesterol, LDL cholesterol, and other relevant variables in patients with type 2 diabetes. The results indicated that each of the aforementioned variables had a varying degree of impact on HbA_1C_ variability. This provides theoretical support for healthcare professionals to implement effective strategies to improve HbA_1C_ variability in diabetic patients, thereby aiding in reducing the detrimental health outcomes associated with this disease and improving the quality of life of patients and their families, as well as minimizing the economic burden on society.

### 4.1 Current status of long-term glycemic variability in patients with T2DM

The results of this study indicated that the HbA_1C_ high variability score (HbA_1C_-HVS) was below the threshold value (60%) reported in the study by Li et al. (19). This was potentially due to the majority of patients being enrolled in outpatient special disease management, which increased their compliance with treatment and resulted in a relatively low HVS. In contrast, the HbA_1c_ standard deviation (HbA_1C_-SD) and coefficient of variation (HbA_1C_-CV) values were higher than those reported in the study by Sun et al. (31), who employed interventions to strengthen glycemic control and reduce glucose fluctuations. However, these values were lower than those reported in the study by Mo et al. (32) on the percentage of glucose variation in Chinese diabetic patients, with a coefficient (%CV) threshold value of 33%. These results suggest that the HbA_1C_ fluctuations observed in this study were relatively stable, and that lower HbA_1C_ variability in diabetic patients could potentially reduce or delay the occurrence of complications associated with diabetes (33). Moreover, lower levels of HbA_1C_ variability in patients could potentially reduce the number of hypoglycemic events, alleviate economic and psychological stress, and improve their quality of life (27).

### 4.2 Risk factors for long-term glycemic variability in patients with T2DM

The present study employed structural equation modeling to investigate the predictors of long-term glycemic variation in patients with type 2 diabetes mellitus. The results indicated that outpatient special disease management, baseline HbA_1c_, mean total cholesterol (TC), and mean triglycerides (TG) had significant direct effects on long-term glycemic variation. Moreover, outpatient special disease management was found to have an indirect effect on long-term glycemic variation by influencing both baseline and recent HbA_1c_. However, long-term glucose variance was not significantly predicted by variance in low-density lipoprotein (LDL), uric acid (UA), recent HbA_1c_, or TC variance. Nevertheless, LDL variance and UA variance were found to have an indirect effect on long-term glucose variance through their effect on mean TC, while TC variance was also found to have an indirect effect on long-term glucose variance through its effect on mean TG.

Diabetes is a persistent, lifelong condition that necessitates frequent medication and monitoring of blood glucose levels, as well as regular assessments for worsening and complications to allow for timely intervention. Patients who meet the requisite criteria for diabetes management are encouraged to participate in outpatient special disease treatment as the diabetes management system improves. The proportion of patients in this study who were enrolled in the diabetes clinic’s special disease management program (69.92%) was higher than that reported by Luo Xiaolu et al. (34) for community-based diabetic patients in Chengdu (48.94%), possibly due to patients’ recognition of the importance of standardized diabetes management. Outpatient management of specific diseases can not only enhance patient health status, functional mobility, and combat obesity (35), but also improve glycemic control and reduce negative outcomes (36), shorten patient stays (37), and reduce medication usage and expenses (38), thereby reducing the financial burden on patients. Additionally, outpatient special disease management can improve patients’ blood glucose control, adherence to treatment, and decrease the incidence of adverse events (39). Greater adherence by patients results in better blood glucose control and timely detection of abnormal values, leading to timely intervention and hence reducing blood glucose fluctuations, ultimately leading to lower HbA_1c_ levels (40), and in turn, reducing long-term bloating. Long-term blood glucose fluctuation can be minimized as a result. A study by Gill et al. (41) on the connection between healthcare expenses and glycemic control in diabetic patients revealed that diabetic patients managed by an endocrinologist specialist had lower healthcare costs, particularly those linked to microvascular and macrovascular complications, and improved HbA_1c_ levels (41). Thus, eligible patients should be encouraged to participate in outpatient special disease care, which will not only minimize treatment expenses and family and societal obligations, but also improve glycemic control and diabetes outcomes, thereby improving patients’ quality of life.

Diabetes is a progressive and insidious condition, which may persist for a period of 4 to 6 years before its initial diagnosis (42). The presence of one or more complications highlights the urgency of early detection and management of glycemic aberrations, which can potentially minimize the severity and incidence of these complications. The baseline level of glycated hemoglobin (HbA_1c_) serves as a pivotal determinant of glucose-lowering therapies (43) and strongly correlates with HbA_1c_ variability (44). Elevated baseline HbA_1c_ levels significantly augment HbA_1c_ decline following therapeutic interventions, precipitating increased variability (45). This variability escalates the risk of patient-related complications and mortality (46). Hence, it is imperative to fortify diabetes education programs and emphasize routine blood glucose monitoring, aimed at detecting and intervening early to minimize blood glucose fluctuations and improve HbA_1c_ variability, thereby impeding the progression of complications and enhancing diabetes-related outcomes.

The present investigation also determined that lipid variability was strongly associated with HbA_1c_ variability. Increased lipid oscillations have been linked to adverse outcomes such as cardiovascular disease and all-cause mortality (47, 48), as well as diabetic complications such as retinopathy, nephropathy, and neurological disease (49, 50). Moreover, the incidence of diabetes and lipid variability were positively correlated (51), suggesting that greater lipid variability is linked to an elevated risk of HbA_1c_ abnormalities. This may be due to the lipotoxic effect of lipids on beta cells, which alters the structure of glucose-activating enzymes and lipid rafts on the cell membrane, leading to beta cell apoptosis (52). Consequently, insulin secretion is reduced and glucose oxidation and utilization are increased, further exacerbating HbA_1c_ variability (53). According to previous research, dyslipidemia plays a pivotal role in the etiology and pathophysiology of diabetes and is strongly positively correlated with HbA_1c_ (54–57). Elevated lipid levels enhance variability by causing beta cell dysfunction and apoptosis (52), leading to reduced insulin secretion and elevated HbA_1c_ levels (56, 58). In addition, uric acid fluctuations have an indirect impact on HbA_1c_ variations. Uric acid is involved in lipid regulation and is positively correlated with cholesterol (59), which can cause dyslipidemia and poor glucose tolerance by inducing hypothalamic inflammation and gliosis and reducing hypothalamic endocrine capacity (60). This leads to suboptimal lipid markers and elevated blood glucose levels, which combined contribute to poor glycemic control and HbA_1c_ oscillations. Uric acid variability has been linked to diabetes (61), cardiovascular risk (62), renal function, and all-cause mortality (63). Thus, elevated levels of lipids and uric acid can impact not only HbA_1c_ variability but also the severity of adverse outcomes and quality of life in diabetes patients. Improvements in the monitoring of lipid and uric acid levels in diabetic patients are necessary for early detection and management to enhance diabetes outcomes. Elevated levels of lipids and uric acid exert not only direct but also indirect effects on the variability of HbA_1C_, contributing to the exacerbation of adverse outcomes and diminished quality of life in individuals with diabetes. The management strategy for glycemic control in diabetic patients revolves around addressing the three principal components of glycemic dysregulation, namely chronic hyperglycemia, hypoglycemia, and glycemic variability. Heightened variability in HbA_1C_ has been associated with numerous unfavorable prognostic factors in diabetes and currently stands as the strongest indicator of diabetic outcomes, rendering it of considerable clinical significance. Hence, early intervention to regulate blood glucose levels and maintain glycemic stability is imperative to enhance patient prognosis and improve their quality of life.

This study did not uncover a significant association between HbA_1C_ variability and changes in follow-up duration. Upon thorough examination of the existing literature, it is evident that no study has thus far provided a specific threshold denoting the extent of risk posed by fluctuations in glycosylated hemoglobin levels for patients. While one study identified a correlation between prolonged variability in glycosylated hemoglobin and cardiovascular disease as well as mortality in individuals with diabetes, the long-term ramifications remain uncertain. Consequently, it is recommended to conduct follow-up investigations spanning a duration of 10 years or more, which may serve as a potential avenue for future research endeavors.

### 4.3 Limitations

This study possesses certain inherent limitations. Primarily, it is a retrospective study conducted at a single medical center, potentially resulting in an inadequate sample size. Secondly, it neglects to incorporate the influence of medications on long-term glycemic variation. It is anticipated that future investigations will employ multi-center surveys, expand the sample size, and undertake thorough exploration of the specific impact of medications on prolonged glucose variability in a prospective study. These endeavors will serve to augment the study’s depth and furnish a theoretical foundation for improved clinical treatment options, thereby enhancing the quality of care provided to patients.

## 5 Conclusions

Using structural equation modeling, we conducted an assessment of the factors that impact HbA_1c_ variability in individuals with type 2 diabetes. Our findings revealed that outpatient special disease management, baseline HbA_1c_, lipids, and uric acid are significant risk factors for HbA_1c_ variation. Consequently, it is recommended that health education programs be intensified for diabetic patients and those at risk, with emphasis on promoting treatment compliance and timely regulation of potential risk factors such as lipids and uric acid to maintain blood glucose stability. This approach is conducive to reducing HbA_1c_ variability, improving adverse outcomes and mortality in diabetic patients, enhancing their quality of life, and reducing the economic burden on families and society.

## Data availability statement

The raw data supporting the conclusions of this article will be made available by the authors, without undue reservation.

## Ethics statement

Written informed consent was not obtained from the individual(s) for the publication of any potentially identifiable images or data included in this article.

## Author contributions

YG, MC, ZX and HQ have determined the topic. LK, JW, and YP collected and organized data. XW, JZ, and XF did data analysis. YG and MC did manuscript writing and revision. ZX and HQ provided advice on draft revisions and finalized them. All authors contributed to the article and approved the submitted version.
